# Favourable outcome of acute hepatitis E infection in patients with ANCA-associated vasculitis

**DOI:** 10.1186/s13023-022-02586-1

**Published:** 2022-12-13

**Authors:** Markus Zeisbrich, Sarah Wendel, Stephanie Finzel, Reinhard E. Voll, Nils Venhoff

**Affiliations:** Department of Rheumatology and Clinical Immunology, Medical Center – University of Freiburg, Freiburg, Germany

**Keywords:** Hepatitis E infection, ANCA-associated vasculitis, Autoimmune disease

## Abstract

**Background:**

Hepatitis E virus (HEV) infection is a frequent cause of acute viral hepatitis. Immunocompromised patients are at increased risk for viral infection and chronic courses of hepatitis. Whether patients with autoimmune diseases are at risk of developing clinically relevant hepatitis or even chronic liver disease after HEV infection is discussed controversially. ANCA-associated vasculitis is a rare autoimmune disease with potentially life-threatening organ involvement, thus requiring intensive immunosuppression with glucocorticoids, cyclophosphamide, or rituximab. As there are no reports available on the infection with HEV in patients with ANCA-associated vasculitis, clinical decision making in such cases is based on experiences from other disease entities. Therefore, in this study we analyzed the course of liver disease and the therapeutic management of autoimmune vasculitis in a retrospective cohort of five patients with ANCA-associated vasculitis and acute hepatitis E.

**Results:**

Four patients were on immunosuppressive maintenance therapy and one patient was on remission induction therapy with cyclophosphamide and high dose glucocorticoids. All patients had at least one potentially hepatotoxic co-medication at the time of hepatitis. Hepatitis-associated clinical symptoms were recorded in four of five patients. The course of hepatitis was characterized by strongly elevated transaminases, a temporary liver failure was observed in one case. The management of hepatitis E included cessation of the immunosuppressants in all patients, whereas oral glucocorticoids were not discontinued. Under this regime, all patients cleared the virus without additional anti-viral treatment. Liver enzymes normalized one month after they peaked. In the follow-up period of at least 1.5 years (range 1.5–12 years), no chronic liver disease was observed, although one patient died of cholangiocarcinoma with liver metastases some years after HEV infection. Vasculitis was not active in our patient cohort at the time of HEV infection. However, inflammatory flares occured in three of five patients after discontinuation of the immunosuppressive therapy. Immunosuppressants were paused for a median time of 4 weeks and after their resumption vasculitic disease activity was controlled in all patients.

**Conclusions:**

Acute HEV infection in patients with ANCA-associated vasculitis shows a favorable outcome of liver disease but bears the risk of inflammatory flares due to cessation of immunosuppression.

## Background

Hepatitis E virus (HEV) is a non-enveloped, single-stranded RNA virus belonging to the Hepeviridae family [1]. Infection with HEV mainly occurs enteric via contaminated water or food. HEV infection is pandemic, also in high-income countries, and one of the most common causes of acute viral hepatitis worldwide [2]. In Europe, 5–15% of acute hepatitis cases are caused by HEV infection. A high prevalence of anti-HEV antibodies in European blood donors between 1 and 52% suggests that HEV is causing several cases of subclinical HEV infection in Europe [3].

The disease course of acute HEV infection is either subclinical or it is leading to self-limiting hepatitis. In immunocompromised patients, HEV infection can become chronic with the risk of the development of liver cirrhosis. This is reported in circumstances of severe immunodeficiency like AIDS, organ transplantation, and hematological malignancies [4–6]. For a decade, patients with inflammatory rheumatic diseases are increasingly treated with potent immunosuppressants by rheumatologists. Whether patients with autoimmune diseases under immunosuppressive therapy are at risk of developing clinically relevant hepatitis or chronic liver disease is discussed controversially [7, 8]. Accordingly, therapy guidelines for patients with autoimmune diseases and acute HEV infection are lacking. Clinical decisions in such cases are based on experience and case reports on a case-to-case basis. Available reports are limited to highly prevalent autoimmune diseases, such as rheumatoid arthritis [9]. In rare autoimmune diseases like ANCA-associated vasculitides (AAV) no reports are available on the infection with HEV and the course of liver disease. AAV are systemic inflammatory diseases with potentially life-threatening organ manifestations especially when lungs and kidneys are involved. Therefore effective treatment usually requires intensified immunosuppression with glucocorticoids, cyclophosphamide, or rituximab, affecting both the humoral and cellular immune response. This results in an amplified risk of overt viral infections and disturbed virus clearance. We analyzed the course of HEV infection in a retrospective cohort of five patients with ANCA-associated vasculitis and acute hepatitis E.

## Results

### Patient characteristics

The main characteristics of patients are provided in Table 1. All but one patient were male and their median age at HEV infection was 57 years (range 36–71). Only one patient (#3) showed frequent consumption of alcohol and none of them had pre-existing chronic liver disease. Two patients (#3, 5) were overweight (BMI > 25). In total, of five vasculitis patients, two had ANCA-negative eosinophilic granulomatosis with polyangiitis (EGPA) and three had granulomatosis with polyangiitis (GPA) with antibodies against proteinase-3. The patients show a wide range of vasculitic organ manifestations typical for the character of the disease, ranging from frequent ear, nose, and throat (ENT; n = 4) involvement to severe organ manifestations like pulmonary (PUL; n = 2) or renal (REN; n = 1) disease. The median time from AAV diagnosis to infection with HEV was 15 months (range 2 months–4 years and 11 months).

**Table 1:** Main demographics and clinical characteristics of patients with both ANCA-associated vasculitis and hepatitis E virus infection

ID	Age (years)	sex	BMI (kg/m^2^)	Alcohol	Vasculitis type	ANCA	Vasculitic organ mani-festations	AAV disease status at HEV infection	BVAS at HEV infection	Time AAV diagnosis to HEV infection (months)	HEV infection symptoms	Hospitali-sation	Follow-up time after HEV infection (years)
#1	51	Male	21.6	None	EGPA	neg	ENT, PUL, PNS, CNS, CAR	Inactive	0	15	Epigastric pain Weight loss Headache Fatigue Light colored Stools Dark colored urine	6 days	5
#2	36	Female	23.0	Rare	EGPA	neg	ENT, PUL, MUS, CUT	Inactive	0	81	Epigastric pain Headache	7 days	2
#3	71	Male	25.4	1 beer per day	GPA	PR3	ENT, REN, CNS	Inactive	0	179	Dermal and scleral icterus Jaundice Light colored stools Dark colored urine Nausea Vomiting	8 days	1.5
#4	58	Male	24.1	None	GPA	PR3	ENT, PNS, CUT	Inactive	0	2	None	4 days	12
#5	57	Male	31.5	None	GPA	PR3	PNS, MUS	Inactive	0	4	Fatigue	no	5

ANCA, anti-neutrophil cytoplasmic antibody; BVAS, Birmingham Vasculitis Activity Score; CAR, cardiac; CNS, central nervous system; CUT, cutaneous; EGPA, eosinophilic granulomatosis with polyangiitis; ENT, ear, nose, and throat; GPA, granulomatosis with polyangiitis; HEV, hepatitis E virus; REN, renal; MUS, musculoskeletal; PNS, peripheral nervous system; PUL, pulmonary

### Immunosuppressive treatment

At the time of infection with HEV, all patients were under immunosuppressive therapy (Table 2). Four patients were on maintenance therapy with either methotrexate (#1, 2, 5) or mycophenolate mofetil (#3). One EGPA patient on methotrexate medication (#1) additionally had mepolizumab for maintenance therapy, a monoclonal antibody against interleukin-5, for his eosinophilic vasculitis. This patient was the only one not having a daily dose of oral prednisolone. The median oral prednisolone dose in reported patients was 5 mg (range 0 – 12.5 mg).

**Table 2:** Immunosuppressive treatment and potentially hepatotoxic medication

ID	Immunosuppressive treatment at time of HEV infection	Cumulative dose of CYC before HEV infection	Cumulative dose of RTX before HEV infection	Daily prednisolone dose at HEV infection	Other potentially hepatotoxic co-medication	Length of immune-suppressant discontinuation	Flare and BVAS	GC increase
#1	MTX 20 mg/week s.c., Mepolizumab 300 mg/month	6 g	0 g	0 mg	Amlodipine	4 months 4 weeks	No	No
#2	MTX 15 mg/week p.o	0 g	0 g	2.5 mg	None	10 weeks	Yes 4	Yes 5 mg
#3	MMF 2 g/day	2 g	2 g	5.0 mg	Trimethoprim/sulfamethoxazole	4 days	Yes 10	Yes 7.5 mg
#4	CYC 15 mg/kg i.v. pulse for remission induction	2.25 g	0 g	12.5 mg	Ciprofloxacin	4 months	No	No
#5	MTX 15 mg/week s.c	0 g	0 g	5 mg	Cefuroxime	4 weeks	Yes 4	No

CYC, cyclophosphamide; HEV, hepatitis E virus; MMF, Mycophenolate mofetil; MTX, methotrexate; p.o., per os; RTX, rituximab; s.c., subcutaneous; i.v., intravenous

Two of the patients on maintenance therapy received cyclophosphamide for remission induction in the past (#1, 3) with one of them having had rituximab in addition (#3). The last administration of cyclophosphamide was four years or one year (patients #1 and #3 respectively) before HEV infection. The last administration of rituximab was ten months (patient #3) before hepatitis E.

In contrast, one patient (#4) was on remission induction therapy with cyclophosphamide and high dose glucocorticoids. This patient received three cycles of cyclophosphamide with a cumulative dose of 2250 mg and a cumulative dose of 1335 mg prednisolone since AAV diagnosis 2 months before.

### Potentially hepatotoxic co-medication

All patients with hepatitis E had at least one potentially hepatotoxic co-medication (Table 2). In three patients (#1, 2, 5), it was the immunosuppressant methotrexate that beard relevant hepatotoxicity. Current or very recent hepatotoxic antibiotic intake coincided with manifest hepatitis in three patients (#3, 4, 5) and patient #1 was on potentially hepatotoxic amlodipine for hypertension treatment. Of note, mepolizumab, mycophenolate mofetil, and cyclophosphamide were not considered to cause clinically apparent acute liver injury.

### Clinical and laboratory presentation of HEV

Four of five patients reported hepatitis-associated clinical symptoms, one patient was asymptomatic (#4). Symptoms included epigastric pain (n = 2), light-colored stools and dark colored urine (n = 2), headache (n = 2), and fatigue (n = 2). Single patients also reported weight loss and nausea with vomiting (Table 1).

Serum concentrations of immunoglobulins IgG, IgA, IgM, differential blood cell counts, and liver tests before, at, and after HEV infection are provided in Table 3. None of the patients had immunoglobulin deficiency at the time of HEV infection, defined as IgG < 7 g/L, IgA < 0.7 g/L, or IgM < 0.4 g/L. Between the three different timepoints there were no statistical significant differences for immunoglobins and differential blood cell counts, although lymphocytes were slightly lower than the reference range at and after HEV infection.

**Table 3:** Mean serum concentrations of immunoglobulins, differential blood cell counts, and liver tests before, at, and after HEV infection

	Normal range	Timepoint 1	Timepoint 2	Timepoint 3	
		Before HEV infection	At HEV infection	4 months post-infection	
IgG (g/l)	7–16	9.16 ± 1.01	9.68 ± 1.0	9.19 ± 0.76	n.s
IgA (g/l)	0.7–4.0	2.14 ± 0.17	2.36 ± 0.38	2.30 ± 0.12	n.s
IgM (g/l)	0.4–2.3	0.81 ± 0.17	1.21 ± 0.34	0.72 ± 0.15	n.s
Leukocytes (× 10^3^/µl)	4.0–10.4	11.07 ± 4.1 H	7.84 ± 0.73	7.36 ± 1.17	n.s
Neutrophils (× 10^3^/µl)	1.9–7.3	8.71 ± 3.99 H	4.96 ± 0.79	5.40 ± 1.21	n.s
Lymphocytes (× 10^3^/µl)	1.2–3.6	1.28 ± 0.18	0.94 ± 0.11 L	1.14 ± 0.23 L	n.s
Monocytes (× 10^3^/µl)	0.25–0.85	0.62 ± 0.09	0.73 ± 0.18	0.64 ± 0.14	n.s
Eosinophils (× 10^3^/µl)	0.03–0.44	0.40 ± 0.30	0.06 ± 0.04	0.10 ± 0.05	n.s
GPT (U/I)	10–50	24.75 ± 4.57	2612 ± 621 H	59.4 ± 14.6 H	1 vs 2 ** 1 vs 3 n.s 2 vs 3 **
GOT (U/I)	10–50	24.80 ± 3.95	1475 ± 364 H	34.40 ± 6.43	1 vs 2 ** 1 vs 3 n.s 2 vs 3 **
γGT (U/I)	< 60	38.20 ± 14.52	318 ± 118 H	35.20 ± 9.34	n.s
Total bilirubin	< 1.4	0.3 ± 0.04	6.24 ± 2.43 H	0.25 ± 0.04	n.s

H, higher than reference range; HEV, hepatitis E virus; L, lower than reference range; n.s., no significant statistical difference; GPT, glutamate-pyruvate transaminase or alanine aminotransferase (ALT); GOT, glutamic-oxaloacetic transaminase or aspartate aminotransferase (AST); γGT; gamma-glutamyl transferase. Data presented as mean (± SEM). *P < 0.05; **P < 0.01. “1 vs 2”, statistical results for timepoint 1 vs timepoint 2

**Fig. 1:**
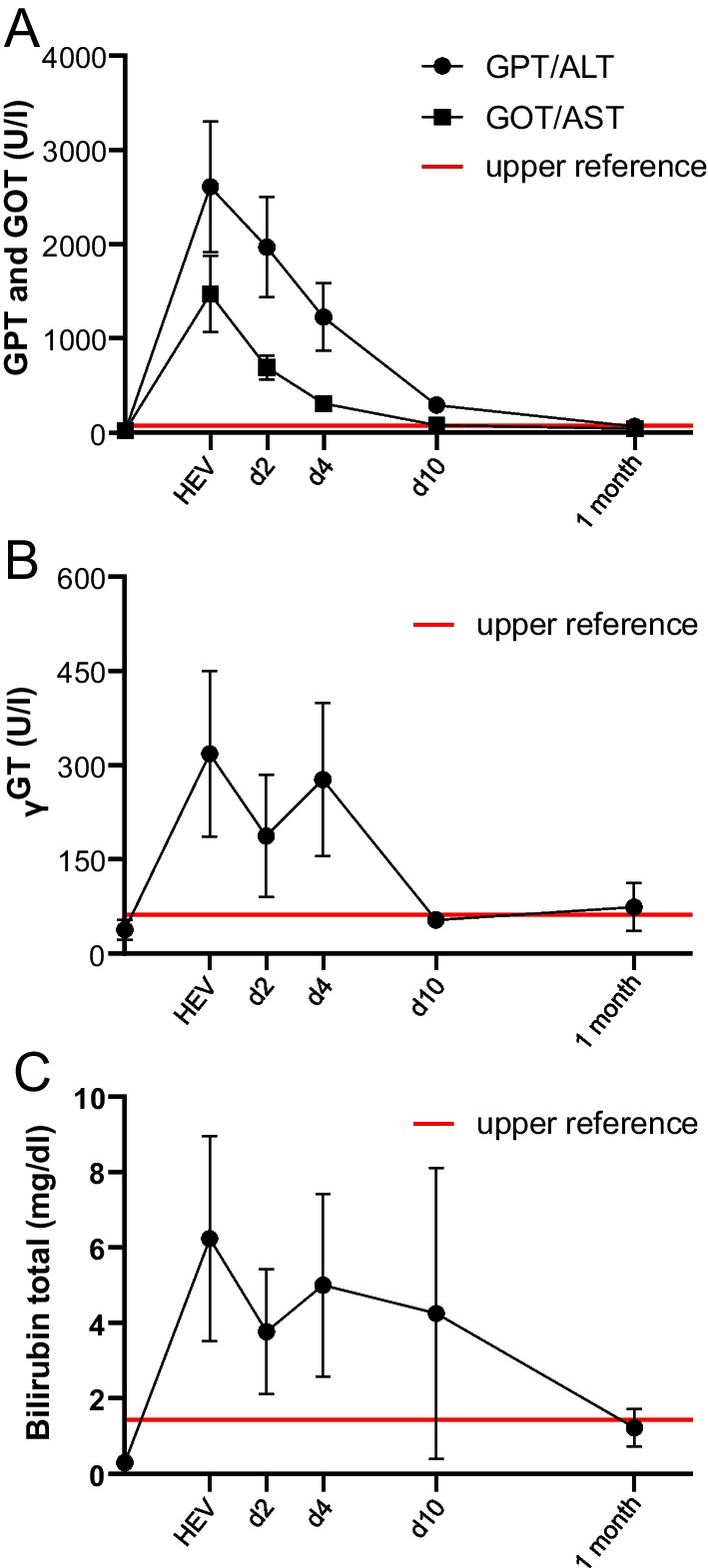
Mean liver enzymes of five AAV patients with active HEV infection over the course of time. Shown are key laboratory values (mean ± SEM) at diagnosis of HEV infection, two days later (d2), four days later (d4), ten days later (d10), and one month after infection. Red lines indicate the upper limit of the reference ranges for depicted liver enzymes. GPT, glutamate-pyruvate transaminase or alanine aminotransferase (ALT); GOT, glutamic-oxaloacetic transaminase or aspartate aminotransferase (AST); γGT; gamma-glutamyl transferase

All patients had normal liver values before HEV infection. Liver transaminases GPT and GOT were significantly higher at HEV infection compared to the time before and after infection, respectively (Table 3). A detailed follow-up on liver testing after diagnosis of HEV infection is provided in Fig. 1. The course of acute HEV infection was predominantly cytolytic with leading GPT/ALT (peak value 2612 ± 621 U/L) over GOT/AST (peak value 1475 ± 364 U/L) in all cases. Transaminases then continuously declined and normalized roughly one month after they peaked, although mean GPT values were still slighty above the reference range. Parameters of cholestasis showed a more fluctuating course in the first days after diagnosis of HEV infection. Gamma-glutamyltransferase (γGT) values normalized between day 4 and day 10 after HEV diagnosis, whereas total levels of bilirubin showed great variability between different patients. Mean bilirubin serum concentrations were recorded within the reference ranges after one month. Only in one patient (#4), liver synthesis was impaired as indicated by an increase in International Normalized Ratio (INR) with a peak value of 1.5 (normal < 1.15) at acute hepatitis. The patient had no signs of active bleeding. C-reactive protein levels were tested normal for all patients except patient #4, who was still on remission induction therapy with cyclophosphamide.

### The outcome of HEV infection

All patients had a follow-up period of at least 1.5 years (range 1.5–12 years). Routine testing for HEV-RNA was performed four months after acute HEV infection and was negative for all patients except #2, who was absent at that time point and confirmed to be HEV-RNA negative by PCR at some point later.

In patient #1 transaminases oscillated slightly above the upper limit of the normal reference range but never peaked again, while liver sonography showed a normal result, and clinically there was no evidence of chronic liver disease in the next five years after HEV infection. For patients #2, #3, and #4, liver enzymes were within the reference range and liver sonography showed normal results in the follow-up period.

In patient #5, elevated liver enzymes were detected at a routine check-up one year after HEV infection. After evaluation in the gastroenterology department, a steatosis hepatis was assumed to cause these laboratory alterations. Another year later, liver enzymes and cholestatic parameters were progressively rising and cholangiocarcinoma with liver metastases was diagnosed. Due to tumor progression, the patient died five years after acute HEV infection. Also patient #3 died 1.5 years after HEV infection due to multiple cerebral ischemias as a result of preexisting cardiovascular disease, which we do not interpret in the context of either AAV or HEV infection.

In summary, while patients #1–4 showed no evidence of chronic liver disease, patient #5 died of cholangiocarcinoma and liver metastases.

### Management of vasculitis

Vasculitis was not active in any of the patients at the time of HEV infection with a BVAS score of 0. However, it must be emphasized that patient #4 was still in the remission induction phase under cyclophosphamide bolus therapy and daily prednisolone dose > 7.5 mg/day and had reached remission 6 weeks before HEV infection. At the time of HEV infection immunosuppressants were discontinued in all patients, oral glucocorticoids were unchanged.

In two patients (#1 and #4), the AAV disease status was unchanged after discontinuation of their immunosuppressants. In one case (patient #1), mepolizumab was continued after four weeks without any evidence of increased disease activity. Methotrexate was paused for a longer period due to patient’s concerns existing already before HEV infection. In the other case (patient #4), cyclophosphamide pulse therapy for remission induction was continued after virus clearance four months later. Within these four months, tapering of oral glucocorticoids was continued without signs of increased vasculitic activity.

In three patients (#2, 3, 5) an increase in disease activity (BVAS scores 4, 10, 4 respectively) was observed after discontinuation of the immunosuppressant. An EGPA patient (patient #2) showed signs of active sinusitis and bronchitis, which were ameliorated by increasing oral prednisolone from 2.5 to 5 mg daily and intensification of inhalative therapy. A GPA patient with ENT involvement (patient #3) developed persistent epistaxis due to a newly diagnosed nasal septum perforation four days after pausing mycophenolate mofetil and his oral glucocorticoids. Mycophenolate mofetil and prednisolone were re-started immediately. In this patient the B cell compartment was still affected by rituximab treatment ten months earlier but he started repopulating peripheral CD19 + B cells (15 cells/µl, 2.9%). To not impair virus elimination, he did not receive another dose of rituximab at this point. In another GPA patient (#5), HEV infection was initially misdiagnosed as methotrexate hepatopathy. Four weeks after treatment discontinuation, the patient himself restarted methotrexate because his arthralgia worsened. Despite restarting methotrexate, liver values had normalized and concerns regarding the diagnosis of a methotrexate hepatopathy led to re-analysis from collected blood samples. HEV-RNA was detected by PCR and recent virus serology indicated a condition after HEV hepatitis.

For all patients, the vasculitic disease was controlled after the resumption of their immunosuppressive treatment. The median time to the re-initiation of the immunosuppressive therapy was 4 weeks (range 4 days–4 months).

## Discussion

In this study, we investigated HEV infection in a cohort of patients with ANCA-associated vasculitis under immunosuppressive therapy. All patients suffered from acute hepatitis and cleared the virus after cessation of immunosuppression. None of the patients needed anti-viral treatment. Three patients received cyclophosphamide before HEV infection: one of them with recent and still ongoing pulse therapy for remission induction in combination with glucocorticoids, and one of them had additional rituximab 10 months before HEV infection. We did not observe prolonged hepatitis or a complicated course of disease in these patients. Still, it has to be acknowledged that the patient with ongoing cyclophosphamide induction therapy, patient #4, was the only one with temporary impaired hepatic function. His INR raised parallel to the increase in transaminases—without clinical signs of subsequent bleeding.

Overall, our observations of the outcome of liver disease after HEV hepatitis in patients with systemic ANCA-associated vasculitis are comparable to those reported in patients with other inflammatory rheumatic diseases, mostly inflammatory arthritides, in which less potent immunosuppressive regimens are common. Chronic hepatitis or fulminant liver failure are very rare after HEV infection in patients with inflammatory arthritides [7, 10]. Our study shows that this is also true for systemic vasculitis patients with severe organ involvement that were treated with highly potent substances like cyclophosphamide, rituximab or high dose glucocorticoids. This stands in contrast to more severe outcomes in patients after solid organ transplantation or under therapy for hematological malignancies [4–6]. A major difference here could be the intensity, the duration and primarily T-cell directed immunosuppression in patients after organ transplantation.

Inconsistent with previous reports, four out of five vasculitis patients in our study presented with clinical symptoms of hepatitis. In a previous study of 23 patients with inflammatory arthritis, almost half of the patients were asymptomatic [7]. Due to the retrospective character of both studies and the limited number of patients, no causative relations can be established here.

In all vasculitis patients, liver enzymes normalized roughly one month after they peaked (Fig. 1). The course of acute HEV was predominantly cytolytic and peak values of liver enzymes were higher in vasculitis patients than reported for patients with inflammatory arthritis and HEV infection [7]. The patient with the harshest immunosuppressive therapy regime at the time of infection had a temporary liver failure but recovered. Before HEV infection, differential blood cell counts showed that neutrophil numbers were increased (Table 3), which is in line with the pathogenic understanding of ANCA-associated vasculitis [11]. At hepatitic disease manifestation and four months after cured hepatitis, mean lymphocyte values were slightly below the normal range although without statistical significance, a finding comparable to what is known as virus-induced lymphopenia [12]. In addition, immunosuppressive therapy with prednisolone (patients #2–5), MMF (patient #3), and cyclophosphamide (patient #4) is also able to cause lymphopenia. Moreover, we could not detect a deficiency of serum immunoglobulins type IgG, IgA and IgM.

Despite a favorable outcome of the acute liver disease after HEV infection, we observed inflammatory flares of the vasculitis in three of five patients (#2, 3, 5) after discontinuation of the immunosuppressant. Thus, it might not be the acute hepatic disease that bears the largest risk for the patients’ health but instead the increased probability of disease relapses. However, in our cohort, increased disease activity did not result in life-threatening organ manifestations like lungs or kidneys and was efficiently controllable by standard therapy.

Patient #5 died from cholangiocarcinoma just a few years after HEV infection, raising the question of a possible correlation between the two diseases. Hepatitis viruses B [13] and C [14] are implicated in the pathogenesis of cholangiocarcinoma. HEV is suspected to promote hepatocellular carcinoma [15] and also carcinogenesis in general [16], but no specific reports on cholangiocarcinoma are available today. Such reports might be expected in the future, as there is growing awareness of HEV infection, especially in patients with immunosuppressive therapy. Interestingly, a histological study of liver biopsies from immunosuppressed patients reported that the presence of destructive cholangitis is typical for HEV infection [17].

Abnormalities in liver function tests are frequent in the daily rheumatologic practice and are routinely attributed to drug-induced liver injury. Case #5 shows that elevated transaminases were incorrectly assigned to methotrexate medication, emphasizing the need to consider and specifically look for virus hepatitis. These cases illustrate that in addition to the screening for hepatitis A, B, and C that is most often performed, hepatitis E must also be considered. Nevertheless, our study shows that all patients experiencing hepatitis had at least one relevant hepatotoxic medication, either their immunosuppressant (methotrexate for patients #1, #2), an antibiotic that was taken before the start of hepatitis (patients #3, #4), or both (patient #5, Table 2). Patient #3, who developed a marked increase in liver enzymes and severe jaundice also consumed alcohol regularly and was overweight, which may have contributed to hepatitis. Whether the combination of immunosuppression together with a potentially hepatotoxic medication enhances the risk for clinically relevant hepatitis, as seen in most patients of this study, needs to be addressed in further studies.

## Conclusions

The average incidence rate of HEV infection in Europe is 19.2% [18] and this number is probably underestimated. Rheumatological patients are susceptible to viral infections including HEV as they are often immunosuppressed and frequently depend on potentially hepatotoxic medication, e.g. antibiotics or immunosuppressants like methotrexate. In five ANCA-associated vasculitis patients, we observed that HEV infection led to clinical symptomatic hepatitis in four of the cases. Despite potent immunosuppression before infection, all patients cleared the virus after cessation of the immunosuppressant without additional anti-viral therapy. No patient developed chronic liver disease. Pausing the immunosuppression led to inflammatory flares, indicating that the management of hepatitis E increases the risk for vasculitic disease relapses.

## Methods

In this retrospective single-center study, patients with a diagnosis of ANCA-associated vasculitis (granulomatosis with polyangiitis, GPA, or eosinophilic GPA, EGPA) classified according to the new ACR/EULAR classification [19, 20] and acute infection with HEV were included. Vasculitis patients were treated according to European and national guidelines [21, 22].

All patients were recruited at the Department of Rheumatology and Clinical Immunology of the University Medical Center Freiburg, Germany, between January 2009 and October 2021. Patients were analyzed through a retrospective chart review, which was approved by the Institutional Review Board of the University of Freiburg Medical School. Written informed consent was obtained from patients. The patients agreed to have their non-identifiable information used for research and publishing. It was not possible to get written informed consent from deceased patients (n = 2). As patient cases have been analyzed anonymously, this proceeding is usual for retrospective anonymous cohorts and was approved by the local Institutional Review Board. In all patients, HEV infection was diagnosed after ruling out hepatitis A, B, and C virus as well as EBV and CMV infection. Acute HEV infection was diagnosed by either detecting HEV RNA in the serum using polymerase chain reaction (PCR) or with reactive anti-HEV IgM and seroconversion to anti-HEV IgG in combination with the typical clinical course of elevated transaminases.

For all patients, detailed information including age, sex, weight and height, date of clinical symptom onset and final diagnosis, prior medical history, and previous immunosuppressive treatment were collected from medical charts. Clinical presentation and vasculitic activity were evaluated by Birmingham vasculitis activity score (BVAS) and typical hepatitis symptoms and hospitalization period were recorded.

Laboratory studies included HEV testing (serology and PCR), laboratory values of GPT/ALT, GOT/AST, γ-GT, total bilirubin, international normalized ratio (INR) c-reactive protein (CRP), creatinine, serum immunoglobulins G, A, M, differential blood cell counts, testing for ANCA by immunofluorescence and specificity against proteinase 3 (PR3) or myeloperoxidase (MPO) by ELISA. Laboratory values are presented as mean ± SEM if not indicated otherwise.

For statistical analysis, one-way ANOVA with post-hoc Tukey’s multiple comparisons test was performed. All data were analyzed by Prism V.9.1.0 (GraphPad).

## Data Availability

The datasets used and analysed during the current study are available from the corresponding author on reasonable request.

## Abbreviations

AAV: ANCA-associated vasculitides
EGPA: Eosinophilic granulomatosis with polyangiitis
γGT: Glutamyltransferase
GOT/AST: Glutamic-oxaloacetic transaminase/aspartate aminotransferase
GPT/ALT: Glutamate pyruvate transaminase/alanine aminotransferase
GPA: Granulomatosis with polyangiitis
HEV: Hepatitis E virus
